# Atmospheric Turbulence Channel Modeling and Performance Analysis of a CO-ZP-OFDM Coherent Optical Communication System for UAV Air-to-Ground Scenarios

**DOI:** 10.3390/e28060714

**Published:** 2026-06-22

**Authors:** Zheming Zhang, Junbin Lou, Yuanjin Lyu, Fanghui Huang, Dawei Wang, Sixu Lu, Yixin He

**Affiliations:** 1 College of Information Science and Engineering, Jiaxing University, Jiaxing 314001, China; 00199895@stu.zjxu.edu.cn (Z.Z.); 00218144@stu.zjxu.edu.cn (Y.L.); lusixu@zjxu.edu.cn (S.L.); 2 College of Mechanical Engineering, Jiaxing University, Jiaxing 314001, China; 3 College of Artificial Intelligence, Jiaxing University, Jiaxing 314001, China; huangfanghui@zjxu.edu.cn; 4 School of Electronics and Information, Northwestern Polytechnical University, Xi’an 710072, China; wangdw@nwpu.edu.cn

**Keywords:** UAV optical communication, CO-ZP-OFDM, Gamma-Gamma turbulence, MMSE channel estimation

## Abstract

This paper targets the air-to-ground (A2G) data backhaul scenario of UAVs and proposes a communication system based on coherent optical zero-padding orthogonal frequency division multiplexing (CO-ZP-OFDM), which unifies atmospheric turbulence scintillation, pointing errors, and Doppler frequency shift into a composite channel model. The system employs the Gamma-Gamma (GG) distribution to describe turbulence-induced intensity fluctuations, a Gaussian beam truncation model to characterize pointing errors, and a dual-pilot method to estimate and compensate the Doppler frequency offset. Furthermore, on a polarization-time-frequency (PTF) three-dimensional orthogonal grid pilot structure, we derive theoretical mean square error (MSE) expressions for the zero-forcing (ZF) and minimum mean square error (MMSE) estimators, and analyze their MSE characteristics under the proposed pilot model. Simulation results show that, under moderate turbulence, the shrinkage factor of the MMSE estimator yields only about 0.4 dB MSE reduction over ZF at SNR=10 dB, whereas the full receiver pipeline that combines coherence-bandwidth pilot averaging with the MMSE and maximum ratio combining (MRC) equalizer reduces the empirical MSE by approximately 15 dB. The bit error rate (BER) performance tests indicate that, under turbulence-free conditions with ideal channel estimation, the system can reduce the BER below $10^{-4}$ at an SNR of approximately 12 dB. Under strong turbulence conditions with MMSE channel estimation, the SNR cost required to achieve a BER of $10^{-3}$ is approximately 18 dB, which corresponds to a 3 to 5 dB BER gain over the ZF baseline at the same SNR. Further simulation analysis shows that the average pointing loss is highly sensitive to the angular jitter at the 1 km link distance: an angular jitter of 1 mrad incurs about 18 dB of loss, and a sub-mrad pointing stability (i.e., $\sigma_{\mathrm{jit}}<0.062$ mrad) is required to keep the average pointing loss below 1 dB.

## 1. Introduction

### 1.1. Background

In recent years, the low-altitude economy has continued to develop rapidly, and unmanned aerial vehicles (UAVs) have been widely deployed in fields such as reconnaissance and surveillance, emergency rescue, express logistics, and urban infrastructure inspection. With the rise of urban air mobility and new low-altitude vehicles such as electric vertical take-off and landing (eVTOL) aircraft, the demand for low-altitude communication infrastructure has grown rapidly [1,2,3,4]. To support these scenarios, a series of enabling techniques has been investigated, ranging from intelligent offloading and resource allocation for digital-twin-aided vehicular networks [5] to reliable cooperative positioning across multiple aerial and ground nodes, in which outlier suppression plays an essential role [6]. In an integrated air-ground network architecture, UAVs need to transmit high-definition video streams, three-dimensional point clouds, and large-capacity sensing data in real time via cooperative routing protocols, imposing extremely high requirements on the bandwidth and reliability of air-to-ground (A2G) data links [7,8]. For post-disaster scenarios in particular, IoT-enhanced airship and buffer-supported emergency communication architectures have been investigated to maintain link availability when conventional infrastructure is unavailable [9]. Beyond pure communications, joint computation offloading in mixed cloud/vehicular-fog computing systems [10] further enhances computational resource utilization in such dynamic aerial scenarios.

Security and secrecy enhancement in low-altitude intelligent networks with complex obstacle environments have also attracted growing research attention, with recent studies extending to short-packet secure transmission for UAV-RIS-assisted links under finite blocklength constraints [11,12,13]. At the network level, near-space communication backbones, satellite-assisted low-altitude access, and federated learning-based ISAC networking over cohesive clustered satellites have been studied to address the high-speed data-backhaul bottleneck across heterogeneous datasets and systems [14,15,16], while digital-twin-assisted edge offloading for low-altitude wireless sensor networks extends these techniques to aerial data backhaul [17]. With the rapid development of UAV integrated sensing and communication (ISAC), RIS-assisted ISAC frameworks, security-aware multi-UAV task offloading, sensing-assisted secure beamforming for RIS-enabled ISAC, RIS-empowered AAV-aided RSMA secure transmission, and integrated resource collaboration for RIS-empowered digital-twin networks have collectively emerged as important research thrusts [18,19,20,21,22,23]. Against this backdrop, the design of new A2G data links for complex mission scenarios has become a central research direction in this field [24].

Compared with traditional microwave communication, free-space optical (FSO) communication offers advantages such as ultra-high bandwidth (>10 Gbps), extremely low probability of interception, and unlicensed spectrum [25,26], with no need to apply for radio spectrum licenses, flexible deployment, and relatively low cost. Andrews and Phillips established the theoretical framework for laser beam atmospheric propagation [27], and related surveys indicate that FSO technology can provide transmission capacity for next-generation high-speed data links that is difficult to achieve with radio frequency communication [28]. Research on FSO technology for beyond-5G applications has shown its significant advantages in spectral efficiency and link capacity [29]. In addition, studies on hybrid FSO/RF systems have further extended the application scope of optical communication in complex atmospheric environments [30]. However, A2G FSO links face three main types of physical impairments. First, random fluctuations of the atmospheric refractive index cause random scintillation in the received optical intensity, severely affecting link reliability [31,32]. Second, random pitch and yaw jitter of the UAV body causes the transmitted beam to deviate from the center of the receiving aperture, resulting in non-negligible pointing-error-induced power loss [33,34]. Third, the Doppler frequency shift produced by high-speed UAV flight at the optical carrier frequency is approximately tens of thousands of times that of a same-speed RF system, posing severe challenges to system phase synchronization [35,36]. The composite effects of these three impairments jointly constrain the quality of UAV FSO communication, and research on efficient and reliable transmission for UAV-assisted relay scenarios has thus become a central research thrust in this field [37].

### 1.2. Related Work and Motivation

In terms of modulation technology, Shieh et al. were the first to propose the coherent optical orthogonal frequency division multiplexing (CO-OFDM) system framework, and verified its strong tolerance to frequency-selective channels and flexible adaptive modulation capability [38,39]. After introducing CO-OFDM into FSO systems, researchers have successively verified the full-duplex FSO transmission characteristics based on hybrid high-order modulation formats, demonstrating good spectral efficiency and BER performance [40,41]. Conventional CO-OFDM uses a cyclic prefix (CP) as the guard interval, whereas the zero-padding (ZP) structure can provide sufficient multipath protection without introducing redundant transmit energy [42], and its spectral efficiency advantage is more prominent in highly dynamic A2G FSO scenarios.

In terms of atmospheric turbulence channel modeling, the Gamma-Gamma (GG) distribution was systematically derived by Al-Habash et al. Its two parameters are uniquely determined by the Rytov variance, and it is applicable to weak, moderate, and strong turbulence regimes simultaneously, making it the mainstream solution for FSO channel modeling [27,31]. The scintillation index is widely used to quantitatively describe the degree of intensity fluctuation, and the aperture-averaging effect can statistically smooth turbulence-induced scintillation at the receiver, serving as an important means of improving link robustness [32,43]. In recent years, novel statistical models such as the inverse Gamma-Gamma distribution and generalized turbulence channels have been proposed to cover a wider range of turbulence regimes [44,45]. In multi-hop relay scenarios, the system performance under the joint effects of doubly inverse Gamma-Gamma turbulence channels and nonzero-boresight pointing errors has also been studied in depth [34,46].

In terms of pointing errors, Farid and Hranilovic established the classical Gaussian beam truncation model, which maps the standard deviation of angular jitter to the average power loss and provides a concise closed-form expression [33]. Studies have shown that, under strong atmospheric turbulence conditions, the use of strong modulation formats such as orthogonal phase-shift keying helps mitigate the joint effects of pointing errors and turbulence-induced scintillation [41]. For aeronautical FSO links, adaptive optics technology has been introduced to actively correct wavefront distortion, and the integration of dynamic beam steering methods has further improved link alignment accuracy [47,48]. Performance analysis of multi-UAV-assisted FSO relay systems has also received extensive attention, with researchers systematically evaluating the BER and outage probability under different turbulence conditions [37].

In terms of Doppler effects and phase synchronization, the Doppler frequency shift at the optical carrier frequency is approximately tens of thousands of times that of a same-speed RF system, posing a serious threat to OFDM subcarrier orthogonality [35,36]. Uncompensated Doppler frequency offsets will destroy inter-subcarrier orthogonality and induce inter-carrier interference (ICI), while the ZP structure exhibits good protection properties in such highly dynamic scenarios [42,49]. Zhang et al. proposed a flexible phase synchronization scheme combining fractionally-spaced blind equalization with an adaptive Kalman filter [35], and Lv and Hong studied an adaptive threshold decision method based on a self-pilot tone to improve demodulation performance under strong fading conditions [43]. Shao et al. systematically studied the signal characteristics of hybrid 16PSK/256QAM-OFDM downlink transmission in full-duplex FSO access systems [40].

In terms of channel estimation, the pilot-based minimum mean square error (MMSE) estimator has been widely studied for its noise-suppression property. By introducing a noise-variance regularization term, it mitigates the noise amplification of the zero-forcing (ZF) estimator under low SNR conditions [49,50]. In recent years, deep learning methods have been widely introduced into FSO channel estimation: Lapsiwala and Vasava applied LSTM neural networks to channel state tracking and prediction over atmospheric turbulence links [51], Kavitha et al. proposed advanced deep learning estimation modules for lognormal turbulence channels [52]. Senthilkumar et al. proposed the sparse wavelength-aware learning network (SWALNet), achieving robust estimation of FSO channels under complex turbulence conditions [53], while Prasanna and Das systematically evaluated the performance of various machine learning methods in FSO channel estimation [54]. Chen et al. established a joint estimation model for FSO channel parameters based on convolutional neural networks [55], and Ahmad et al. studied a high-capacity FSO communication scheme based on orbital-angular-momentum structured light and intelligent adaptive signal processing [56]. Ishaq and Jamel proposed a sequence-aware OOK detection method based on bidirectional GRU/LSTM, and verified its performance improvement under strong turbulence scenarios [57].

Although the above works have achieved important progress in their respective fields, several shortcomings remain. First, most existing FSO studies investigate a single impairment mechanism in isolation, lacking systematic modeling of the composite effects of atmospheric turbulence scintillation, pointing errors, and Doppler frequency shift [28,29]. Second, most existing FSO-OFDM systems still adopt the conventional CP guard interval and have not fully exploited the inherent advantages of the ZP structure in multipath protection and spectral efficiency [42]. Third, existing deep-learning-based channel estimation schemes are mostly modeled for a single impairment and have difficulty effectively covering highly dynamic multi-impairment composite scenarios [53,54], and in-depth research on polarization-time-frequency (PTF) three-dimensional pilot design and joint multi-impairment MMSE estimation algorithms for UAV A2G scenarios is still lacking [37,57]. These shortcomings collectively pose significant constraints on the practical reliability of UAV FSO communication in complex dynamic environments.

To explicitly address the three gaps identified above, this paper makes the following targeted contributions. (G1) A hierarchical cascaded composite-channel model is constructed in Section 2.1, jointly modeling the three impairments (turbulence scintillation, pointing-error loss, and Doppler frequency shift) in a unified mathematical framework, in contrast to the single-impairment focus of prior works. (G2) A CO-ZP-OFDM system framework is adopted in Section 2.1, replacing the conventional CP-OFDM guard interval with a zero-padding structure that simultaneously provides multipath protection and improves spectral efficiency under highly dynamic A2G conditions. (G3) A polarization-time-frequency (PTF) three-dimensional sparse pilot grid is designed in Section 3.2, together with a closed-form MMSE estimator with explicitly stated modeling assumptions and coherence-bandwidth pilot averaging, which together deliver the 15 dB MSE gain and 3 to 5 dB BER gain reported in Section 4.2 over the ZF baseline. In addition, the Doppler sensitivity analysis further quantifies the precision requirement of the Doppler tracking algorithm, completing the closed loop from impairment modeling to system-level performance evaluation.

### 1.3. Contributions

Motivated by the above research, this paper proposes a complete communication scheme based on coherent optical zero-padding orthogonal frequency division multiplexing (CO-ZP-OFDM) for the UAV air-to-ground scenario, unifying the three types of impairments into a composite channel model, designing a hierarchical cascaded receiver algorithm, and comprehensively evaluating the performance under different impairment conditions through simulation experiments. The main contributions are as follows:

(1) A composite channel model for the UAV A2G scenario is established. Atmospheric turbulence is statistically modeled using the Gamma-Gamma distribution [27,31], pointing errors are characterized by the Gaussian beam truncation model, and the mapping relationship between the standard deviation of angular jitter and the average power loss is analyzed [33,34], and the Doppler frequency shift is estimated and pre-compensated using the dual-pilot method [35,36]. The three types of impairments are unified into a hierarchical cascaded MIMO channel framework. By decomposing the channel matrix into factors such as turbulence attenuation, pointing loss, frequency-offset phase, and polarization coupling, the joint fading characteristics of signal transmission in dynamic environments are described, providing an accurate and decomposable channel representation for subsequent receiver design.
(2) A channel estimation scheme based on sparse pilots and regularized estimation is proposed. For the proposed CO-ZP-OFDM structure, a polarization-time-frequency (PTF) three-dimensional orthogonal pilot pattern is designed, with a low pilot overhead [49,50]. Closed-form theoretical mean square error (MSE) expressions for the ZF and MMSE estimators under this pilot structure are derived, and the role of the regularization term in suppressing noise amplification at low SNR is analyzed in closed form.
(3) The proposed scheme is evaluated through simulation experiments on dynamic A2G links. The evaluation covers channel statistical characteristics, estimator MSE performance, and bit error rate (BER). Under moderate turbulence and typical mobility, the BER reaches the order of $10^{-3}$ at around 12 dB SNR, with a measurable gain over the conventional ZF baseline.

### 1.4. Paper Organization

The remainder of this paper is organized as follows. Section 2 describes the air-to-ground system model and channel modeling. Section 3 introduces the channel estimation and equalization algorithms. Section 4 presents the simulation parameter settings and result analysis. Section 5 concludes the paper.

## 2. Air-to-Ground Optical Transmission System and Channel Modeling

**Figure 1:**
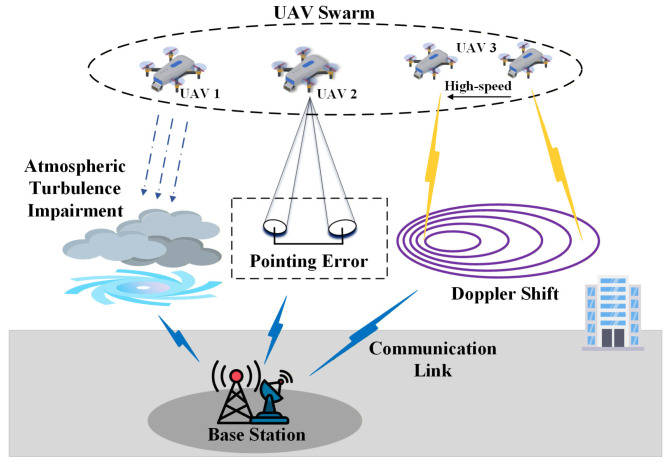
UAV-assisted coherent optical ZP-OFDM system model.

As shown in Figure 1, for the dynamic data backhaul scenario in which a UAV acts as an aerial transmission platform, this work constructs a coherent optical zero-padding orthogonal frequency division multiplexing (CO-ZP-OFDM) communication architecture based on polarization division multiplexing (PDM). This architecture aims to overcome multiple impairments in the A2G link, including intensity scintillation caused by atmospheric turbulence, pointing errors induced by platform micro-vibration, and Doppler frequency shifts produced by high-speed mobility, laying the system foundation for highly reliable optical communication backhaul in highly dynamic scenarios.

### 2.1. Frame Structure and Three-Dimensional Pilot Grid Model

**Figure 2:**
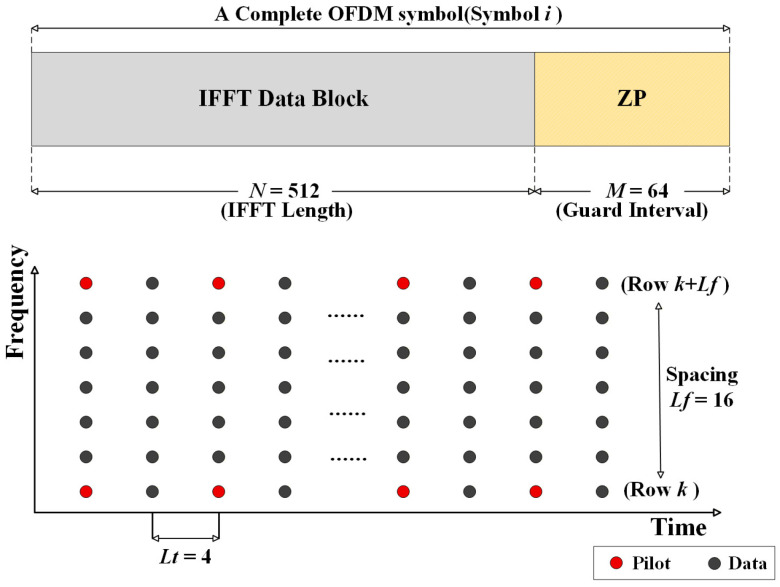
Schematic diagram of the CO-ZP-OFDM frame structure.

To adapt to the characteristics of highly dynamic A2G channels, we redefine the frame structure of the time-domain signal, as shown in Figure 2. Let the time-domain transmit vector of the *l*-th orthogonal frequency division multiplexing (OFDM) symbol on polarization *p* be $x_{l,p}$. This vector is formed by concatenating the effective data payload with the guard interval:

(1) $$x_{l,p}={\left[{\left(F_{N_{\mathrm{FFT}}}^{H}s_{l,p}\right)}^{T},\;0_{M\times 1}^{T}\right]}^{T},$$

where $N_{\mathrm{FFT}}$ denotes the FFT/IFFT size (set to $N_{\mathrm{FFT}}=512$ in this work), $F_{N_{\mathrm{FFT}}}$ is the $N_{\mathrm{FFT}}$-point normalized discrete Fourier transform (DFT) matrix, $s_{l,p}$ is the frequency-domain modulation symbol vector of length $N_{\mathrm{FFT}}$ populated only on the active subcarrier set (the center $N_{\mathrm{sub}}=128$ data subcarriers used in this work, with the remaining $N_{\mathrm{FFT}}-N_{\mathrm{sub}}$ entries set to zero to act as oversampling guards), and $0_{M\times 1}$ represents the zero-padding (ZP) sequence of length M=64 appended after each IFFT block. The total length of one OFDM symbol in samples is therefore $N_{\mathrm{FFT}}+M=576$, with corresponding symbol duration $T_{\mathrm{sym}}=\left(N_{\mathrm{FFT}}+M\right)/\left(N_{\mathrm{FFT}}\;\Delta f\right)=576/\left(512\times 100\times 10^{3}\right)\approx 11.25\;\mu$s. This structure places the effective symbols at the head of the vector and appends the ZP sequence as a guard interval at the tail, providing joint protection against multipath delay and timing errors.

For the choice of key frame parameters, this work sets the guard interval to M=64. This value covers the multipath delay spread caused by atmospheric scattering ($\tau_{\max}\approx 3$ ns) and the timing synchronization errors induced by UAV body jitter ($\Delta \tau_{\mathrm{rms}}\approx 3$ ns), so that inter-symbol interference (ISI) at the physical layer is bounded by the chosen guard window. The guard interval length *M* must satisfy:

(2) $$M\geq \max\left(\left\lceil \tau_{\max}/T_{s}\right\rceil ,\;\left\lceil \Delta \tau_{\mathrm{rms}}/T_{s}\right\rceil \right),\;T_{s}=\frac{1}{N_{\mathrm{FFT}}\cdot \Delta f},$$

where $T_{s}$ is the sampling period of a single subcarrier, and Δf is the subcarrier spacing. Under a typical parameter configuration ($N_{\mathrm{FFT}}=512$, Δf=100 kHz), $T_{s}=1/\left(512\times 100\times 10^{3}\right)\approx 19.5$ ns, so M=64 can provide a guard interval of approximately 1.25 μs, which is much larger than the actually required 3 ns and provides sufficient margin against multipath spread.

In terms of pilot design, the system abandons the traditional comb-type pilots and instead adopts a sparse polarization-time-frequency (PTF) three-dimensional orthogonal grid. Specifically, based on the channel coherence time of $T_{\mathrm{rms}}\approx 10$ ms at a flight speed of 100 km/h, the time-domain step is set to $\Delta t_{p}=4$, which satisfies the Nyquist criterion and reduces redundant overhead by approximately 50% compared with per-symbol insertion. Meanwhile, to match the coherence bandwidth characteristics of the atmospheric turbulence channel, the frequency-domain step is set to $\Delta f_{p}=16$. The three-dimensional orthogonality of the PTF pilots can be expressed as:

(3) $$P_{p}P_{p}^{H}=\alpha_{p}I_{2},\;P_{p}P_{q}^{H}=0\;\left(p\neq q\right),$$

where $\alpha_{p}$ is the pilot power enhancement factor (typically 2 or 4), and $I_{2}$ is the 2×2 identity matrix. This orthogonality condition ensures that the pilot signals between different polarization states and time slots are mutually independent, thereby supporting low-complexity channel estimation algorithm design and avoiding pilot contamination between multiple polarizations.

The pilot overhead ratio η can be calculated as:

(4) $$\eta =\frac{1}{\Delta f_{p}\cdot \Delta t_{p}}.$$

Under a typical configuration ($\Delta f_{p}=16$, $\Delta t_{p}=4$), η=1.56%. Compared with the approximately 9% pilot overhead of conventional schemes, the pilot overhead is reduced by about 7.44 percentage points under the PTF three-dimensional orthogonal grid.

### 2.2. Dynamic A2G MIMO Channel Model

Under the quasi-static assumption, after the overlap-add (OLA) operation at the receiver, the frequency-domain received signal vector at the *l*-th OFDM symbol and the *k*-th subcarrier $y_{l,k}$ satisfies

(5) $$y_{l,k}=H_{l,k}\;x_{l,k}+n_{l,k},$$

where $n_{l,k}∼\mathrm{CN}\left(0,\sigma_{n}^{2}I\right)$ is additive white Gaussian noise (AWGN) with covariance matrix $\sigma_{n}^{2}I$. The core channel transfer matrix $H_{l,k}$ integrates multiple physical impairment mechanisms, decomposed as follows:

(6) $$H_{l,k}=I_{l,k}\cdot \eta_{p}\cdot e^{j2\pi f_{D}lT_{\mathrm{sym}}}\cdot J,$$

where $I_{l,k}$ is the atmospheric turbulence scintillation factor, $\eta_{p}$ is the pointing-error loss, $e^{j2\pi f_{D}lT_{\mathrm{sym}}}$ is the Doppler phase rotation term, and J is the polarization-coupling Jones matrix. The impairment components are mutually independent and can be modeled hierarchically and compensated stage by stage. Specific models for each component are presented in the following subsections.

#### 2.2.1. Atmospheric Turbulence Intensity Scintillation Model

The optical intensity fluctuation *I* caused by atmospheric turbulence follows the Gamma-Gamma statistical distribution [42], and its probability density function (PDF) is given by:

(7) $$f\left(I|\alpha ,\beta \right)=\frac{2{\left(\alpha \beta \right)}^{(\alpha +\beta )/2}}{\Gamma (\alpha )\Gamma (\beta )}\;I^{(\alpha +\beta )/2-1}\cdot K_{\alpha -\beta }\;\left(2\sqrt{\alpha \beta I}\right),\;I>0,$$

where Γ(·) is the Gamma function, and $K_{\alpha -\beta }\left(\cdot \right)$ is the modified Bessel function of the second kind. Parameters α and β characterize the effective number of large-scale and small-scale turbulent eddies, respectively, and jointly determine the statistical characteristics of intensity fluctuation. Their relationship with the refractive index structure constant $C_{n}^{2}$ can be established through the Rytov variance $\sigma_{R}^{2}$:

(8) $$\sigma_{R}^{2}=1.23\;C_{n}^{2}\;k^{7/6}\;L^{11/6},$$

(9) $$\alpha ={\left[\exp\;\left(\frac{0.49\;\sigma_{R}^{2}}{{\left(1+1.11\;\sigma_{R}^{12/5}\right)}^{7/6}}\right)-1\right]}^{-1},$$

and

(10) $$\beta ={\left[\exp\;\left(\frac{0.51\;\sigma_{R}^{2}}{{\left(1+0.69\;\sigma_{R}^{12/5}\right)}^{5/6}}\right)-1\right]}^{-1},$$

where k=2π/λ is the optical wavenumber, and *L* is the transmission distance. For a typical UAV communication scenario (transmission distance L=1 km, wavelength λ=1550 nm, $C_{n}^{2}=10^{-14}$ $m^{-2/3}$), the Rytov variance is calculated as:

(11) $$\sigma_{R}^{2}=1.23\times 10^{-14}\times {\left(\frac{2\pi }{1550\times 10^{-9}}\right)}^{7/6}\times 1000^{11/6}\approx 0.2255.$$

Substituting into Equation (9) yields α≈11.607, β≈10.094. The corresponding scintillation index (SI) is defined as:

(12) $$\mathrm{SI}=\frac{1}{\alpha }+\frac{1}{\beta }+\frac{1}{\alpha \beta }.$$

Substituting the numerical values gives SI≈0.192. Under the described link conditions, the channel is at the weak-to-moderate turbulence boundary, which is consistent with theoretical predictions and typical observations for this scenario. The numerical values used here are identical to those reported in Table 1 and Section 4.1.

The Gamma-Gamma PDF in Equations (9) and (10) describes the turbulence-induced optical intensity fluctuation *I*. Because the proposed CO-ZP-OFDM receiver is coherent and operates on the complex baseband field amplitude, the intensity sample *I* drawn from the Gamma-Gamma distribution is mapped to a complex turbulence channel gain

(13) $$h_{\mathrm{turb}}=\sqrt{I}\;\exp\left(j\phi_{\mathrm{turb}}\right),$$

where $\phi_{\mathrm{turb}}∼U\left[0,2\pi \right)$ is the random optical phase induced by turbulent path-length fluctuations. The square-root transformation maps the intensity scintillation index $\mathrm{SI}=\mathrm{Var}\left(I\right)/E{\left[I\right]}^{2}$ to the field-amplitude scintillation index ${\mathrm{SI}}_{\mathrm{amp}}=\mathrm{Var}(|h_{\mathrm{turb}}\left|\right)/E[|h_{\mathrm{turb}}{\left|\right]}^{2}$, which is the quantity that drives the per-subcarrier fading in the coherent OFDM model of Equation (9) onwards. This $h_{\mathrm{turb}}$ is the multiplicative scalar that enters the polarization-coupling Jones-matrix channel in Section 2.2.4, and is used in all coherent OFDM simulations throughout this paper.

#### 2.2.2. Pointing Error Loss Model

The pointing-error loss caused by random UAV pitch/yaw attitude follows the Beckmann distribution [26]. Assuming that the radial offset of the beam at the receiver *r* follows the Rayleigh distribution, its probability density function is:

(14) $$f\left(r\right)=\frac{r}{\sigma_{r}^{2}}\;\exp\;\left(-\frac{r^{2}}{2\sigma_{r}^{2}}\right),\;r\geq 0,$$

where $\sigma_{r}=\sigma_{\mathrm{jit}}\times L$ is the standard deviation of the linear displacement at the receiving plane, and $\sigma_{\mathrm{jit}}$ is the standard deviation of the angular jitter (typically 1 to 5 mrad). The power attenuation caused by pointing errors can be modeled by the Gaussian beam truncation model:

(15) $$\eta_{p}\left(r\right)=A_{0}\;\exp\;\left(-\frac{2r^{2}}{w_{\mathrm{eq}}^{2}}\right),$$

where $A_{0}={\left[\mathrm{erf}\left(u\right)\right]}^{2}$ is the power attenuation factor when the optical axis is aligned (considering geometric loss and atmospheric absorption), $u=\sqrt{\pi /2}\;a_{\mathrm{recv}}/w_{L}$ is the aperture truncation parameter, and $w_{\mathrm{eq}}$ is the equivalent beam waist radius at the receiver. The beam radius $w_{L}$ is related to the transmission distance by:

(16) $$w_{L}=w_{0}\;\sqrt{1+{\left(\frac{\lambda L}{\pi w_{0}^{2}}\right)}^{2}},$$

where $w_{0}$ is the beam waist radius at the transmitter. For a configuration of $w_{0}=2$ cm (consistent with Table 1), the beam divergence angle is

(17) $$\theta_{\mathrm{div}}\approx \frac{\lambda }{\pi w_{0}}=\frac{1550\times 10^{-9}}{\pi \times 0.02}\approx 24.67\;\mu \mathrm{rad}.$$

At L=1 km, the receiver spot radius is approximately:

(18) $$w_{L}\approx \theta_{\mathrm{div}}\times L=24.67\times 10^{-6}\times 1000\approx 31.76\;\mathrm{mm}.$$

It should be emphasized that $w_{L}$ and $w_{\mathrm{eq}}$ are two distinct quantities. $w_{L}$ is the geometric Gaussian beam radius at the receiver plane, which describes only the diffraction-induced beam expansion. In contrast, $w_{\mathrm{eq}}$ in Equation (15) is the equivalent beam waist radius defined by the Farid-Hranilovic model [33], which incorporates the aperture-truncation effect at the receiver. Under the present configuration ($a_{\mathrm{recv}}=0.15$ m, $w_{L}\approx 31.76$ mm), the aperture is much larger than the beam spot, so the truncation parameter $u=\sqrt{\pi /2}\;a_{\mathrm{recv}}/w_{L}\approx 5.92$ lies in the weak-truncation regime where $A_{0}={\left[\mathrm{erf}\left(u\right)\right]}^{2}\to 1$ and almost all transmitted power is captured in the aligned case. In this regime, the equivalent beam-waist radius is well approximated by the engineering relation

(19) $$w_{\mathrm{eq}}\approx \frac{2}{\sqrt{3}}\;a_{\mathrm{recv}}\;\approx \;1.1547\;a_{\mathrm{recv}},$$

which yields $w_{\mathrm{eq}}\approx 0.1732$ m, identical to the value listed in Table 1. Equations (15) and (20) are evaluated with this $w_{\mathrm{eq}}$.

Taking the expectation of f(r) over *r*, the closed-form expression for the average power loss is:

(20) $$\langle \eta_{p}\rangle =A_{0}\;\cdot \frac{w_{\mathrm{eq}}^{2}}{w_{\mathrm{eq}}^{2}+2\sigma_{r}^{2}}.$$

Substituting $w_{\mathrm{eq}}\approx 0.1732$ m and $A_{0}\approx 1$ into Equation (20), the average pointing loss as a function of $\sigma_{\mathrm{jit}}$ (with $\sigma_{r}=\sigma_{\mathrm{jit}}\cdot L$) is summarized as follows: $\sigma_{\mathrm{jit}}=0.1$ mrad yields a loss of about 2.2 dB, $\sigma_{\mathrm{jit}}=0.5$ mrad yields about 12.5 dB, $\sigma_{\mathrm{jit}}=1$ mrad yields about 18.3 dB, $\sigma_{\mathrm{jit}}=2$ mrad yields about 24.3 dB, and $\sigma_{\mathrm{jit}}=5$ mrad yields about 32.2 dB. The radial displacement $\sigma_{r}$ scales linearly with *L*, so at L=1 km even a 1 mrad jitter produces a 1 m displacement, which is far larger than $w_{\mathrm{eq}}$. Consequently, the pointing-error loss is highly sensitive to the angular jitter, and a sub-mrad pointing stability is required to keep the average loss within a few dB. To bound the average loss within 1 dB, the angular jitter must satisfy $\sigma_{\mathrm{jit}}<0.062$ mrad. This indicates that, contrary to the preliminary impression suggested by very small numerical examples, pointing errors are a major source of link power loss in UAV A2G FSO links and must be jointly considered with atmospheric turbulence in the system design.

#### 2.2.3. Doppler Frequency Shift and Phase Noise

The phase rotation term is mainly caused by the Doppler effect. Let the radial velocity of the UAV relative to the ground station be $v_{r}$ (positive values indicate moving away). The Doppler frequency shift is then:

(21) $$f_{D}=\frac{v_{r}}{\lambda }\;\cos\theta ,$$

where λ is the carrier wavelength, and θ is the angle of arrival. At the *l*-th OFDM symbol instant ($t_{l}=l\cdot T_{\mathrm{sym}}$, $T_{\mathrm{sym}}$ is the symbol period), the accumulated phase is:

(22) $$\varphi_{l}=2\pi f_{D}\;l\;T_{\mathrm{sym}}.$$

For λ=1550 nm wavelength system, when the radial velocity reaches 100 km/h (about 27.8 m/s), the Doppler frequency shift is approximately:

(23) $$f_{D}=\frac{v_{r}}{\lambda }=\frac{27.8}{1550\times 10^{-9}}\approx 17.9\;\mathrm{MHz}.$$

If the OFDM subcarrier spacing is Δf=100 kHz, the Doppler frequency shift corresponds to a deviation of approximately 179 subcarriers, which must be eliminated by accurate frequency-offset estimation and compensation algorithms. The phase change within one symbol period is:

(24) $$\Delta \varphi =2\pi f_{D}T_{\mathrm{sym}}=2\pi \times 17.9\times 10^{6}\times 11.25\times 10^{-6}\approx 403\pi \;\mathrm{rad}.$$

This means that the phase rotates approximately 201 times within each OFDM symbol. Without compensation, this will cause severe inter-carrier interference, and accurate Doppler pre-compensation must be performed at the receiver.

#### 2.2.4. Polarization-Coupling Jones Matrix

The polarization coupling term J is the 2×2 Jones matrix, which describes the energy leakage between the X/Y polarization states caused by non-ideal orthogonality. Considering polarization mode dispersion (PMD) and polarization-dependent loss (PDL) effects, its decomposition is:

(25) $$J=R\left(\theta_{r}\right)\cdot \mathrm{diag}\;\left(\sqrt{g_{1}},\;\sqrt{g_{2}}\right)\cdot R\left(-\theta_{t}\right)⊙e^{j\Phi },\;R\left(\theta \right)=\left[\begin{array}{cc} \cos\theta & -\sin\theta \\ \sin\theta & \cos\theta \end{array}\right],$$

where $\theta_{t}$ and $\theta_{r}$ are the deflection angles of the principal polarization axes at the transmitter and receiver, respectively, and $g_{1}$ and $g_{2}$ are the power gains of the two principal polarization states (satisfying $g_{1}\geq g_{2}>0$), Φ is the frequency-dependent phase response matrix, and ⊙ denotes element-wise multiplication. The diagonal elements characterize the transmission gain of the principal polarization states, while the off-diagonal elements characterize the polarization crosstalk intensity. PDL is defined as the ratio of the two principal polarization-state gains:

(26) $$\mathrm{PDL}=10\log_{10}\;\left(\frac{g_{1}}{g_{2}}\right)\;\left[\mathrm{dB}\right].$$

For a typical FSO-fiber concatenated link, PDL values are usually in the range of 0.5 to 2 dB. If PDL=1 dB, then $g_{1}/g_{2}=10^{0.1}\approx 1.259$. Combined with the normalization condition $g_{1}+g_{2}=2$, we obtain $g_{1}\approx 1.118$, $g_{2}\approx 0.888$, and the polarization gain imbalance is about 12%, which is within the engineering tolerance.

## 3. Channel Estimation and Equalization Algorithm

For the composite-impairment channel model described above, this section designs a hierarchical cascaded channel estimation and equalization algorithm framework. The framework targets the time-varying MIMO channel through three modules: Doppler pre-compensation, minimum mean square error (MMSE) pilot-aided estimation, and adaptive two-dimensional interpolation. The three modules are executed sequentially. First, the Doppler frequency shift is eliminated to restore subcarrier orthogonality. Next, channel matrix estimation is performed on the sparse pilot grid. Finally, the estimation results are extended to all data subcarriers via two-dimensional interpolation, yielding the channel estimates used by the subsequent equalizer.

### 3.1. Doppler Frequency Offset Estimation and Compensation

The carrier frequency offset $f_{D}$ caused by the Doppler effect causes a mismatch in subcarrier orthogonality and induces inter-carrier interference (ICI). Therefore, this frequency shift must be eliminated before channel estimation. This section adopts the dual-pilot method, using two adjacent pilot symbols with a time step of $\Delta t_{p}$ for frequency-offset estimation, which is simple, efficient, and suitable for high-mobility scenarios. Let the received vectors of the *l*-th and the $\left(l+\Delta t_{p}\right)$-th pilot symbols at the *k*-th subcarrier be $y_{l,k}$ and $y_{l+\Delta t_{p},k}$, respectively, with the corresponding pilot matrix denoted as $P_{k}$. Under the assumption that the channel gain remains constant during $\Delta t_{p}$ symbols (quasi-static assumption), we have:

(27) $$y_{l,k}=e^{j\varphi_{l}}\cdot H_{l,k}\cdot P_{k}+N_{l,k}.$$

Using the orthogonality of the pilots, $P_{k}P_{k}^{H}=\alpha_{p}I$, an initial channel estimate (zero-forcing estimate) can be obtained:

(28) $${\hat{H}}_{l,k}^{\left(0\right)}=y_{l,k}\cdot P_{k}^{-1}.$$

Under ideal conditions (no noise, no turbulence fluctuation), the ratio of the estimated values at the two adjacent pilot instants should contain only the phase rotation term, and the channel factor cancels out completely:

(29) $${\hat{H}}_{l+\Delta t_{p},k}^{\left(0\right)}/{\hat{H}}_{l,k}^{\left(0\right)}\approx e^{\;j2\pi f_{D}\Delta t_{p}T_{\mathrm{sym}}}.$$

Therefore, by averaging the angle information over all pilot subcarriers, the Doppler frequency offset estimate is obtained:

(30) $${\hat{f}}_{D}=\frac{1}{2\pi \Delta t_{p}T_{\mathrm{sym}}}\cdot \frac{1}{N_{p}}\sum_{k\in K_{p}}∠\;\left({\hat{H}}_{l+\Delta t_{p},k}^{\left(0\right)}/{\hat{H}}_{l,k}^{\left(0\right)}\right),$$

where $K_{p}$ is the index set of pilot subcarriers, and $N_{p}$ is the total number of pilot subcarriers. Averaging over $N_{p}$ pilot subcarriers reduces the per-subcarrier noise variance by a factor of $N_{p}$ in the angle estimate. After obtaining the frequency-offset estimate, an inverse phase compensation is applied to all received symbols:

(31) $${\tilde{y}}_{l,k}=y_{l,k}\cdot e^{-\;j2\pi {\hat{f}}_{D}lT_{\mathrm{sym}}},$$

This compensation step maps the received signal from the Doppler-shifted frequency grid back to the standard OFDM subcarrier positions, so that the residual frequency offset within the subsequent estimation window is reduced.

Furthermore, the variance of the dual-pilot frequency-offset estimate can be expressed as:

(32) $$\mathrm{Var}\left({\hat{f}}_{D}\right)=\frac{1}{8\pi^{2}\;\cdot \Delta t_{p}^{2}\;\cdot T_{\mathrm{sym}}^{2}\;\cdot N_{p}\;\cdot \mathrm{SNR}}.$$

For $\Delta t_{p}=4$, $T_{\mathrm{sym}}=11.25\;\mu$s, and $N_{p}=32$, at SNR=15 dB, the estimation standard deviation is about 0.11 kHz, which is approximately five orders of magnitude smaller than the typical Doppler frequency shift of 17.9 MHz at this operating point.

### 3.2. MMSE Channel Estimation

Before deriving the estimators, we explicitly state the modeling assumptions used in this subsection so that the closed-form MSE expressions are reproducible and the simplifications behind the final scalar shrinkage form ${\hat{H}}_{\mathrm{MMSE}}=\frac{\mathrm{SNR}}{\mathrm{SNR}+1}{\hat{H}}_{\mathrm{ZF}}$ are transparent. On a single pilot subcarrier *k*, the received signal at the receiver after Doppler compensation is modeled as a scalar relation $y_{p}=hp+n_{p}$, where h∈C is the composite channel coefficient, p∈C is the pilot symbol, and $n_{p}∼\mathrm{CN}\left(0,\sigma_{n}^{2}\right)$ is the AWGN sample. The polarization coupling and full 2×2 Jones matrix are treated in a separate pre-equalization stage and are not part of the estimator derivation in this subsection.

- On the one hand, the pilot design follows an orthogonal structure with unit pilot energy. The polarization-time-frequency pilot sequence satisfies $P^{H}P=E_{p}I$ with $E_{p}=1$ under the normalization adopted throughout this paper. Under this normalization, the least-squares solution at the pilot grid reduces to ${\hat{h}}_{\mathrm{ZF}}=y_{p}/p$ and is unbiased. This formulation enables a direct interpretation of the estimation process while maintaining analytical tractability.
- On the other hand, the channel is modeled with an independent Gaussian prior $h∼\mathrm{CN}(0,\sigma_{h}^{2})$, which is statistically independent of the noise term $n_{p}$. All MSE quantities are reported in normalized form with $\sigma_{h}^{2}=1$, namely ${\mathrm{MSE}}_{\mathrm{dB}}=10\log_{10}\left(\mathrm{MSE}/\sigma_{h}^{2}\right)$. Accordingly, the per-subcarrier pilot SNR is defined as $\mathrm{SNR}≜\sigma_{h}^{2}/\sigma_{n}^{2}$. These assumptions correspond to the standard one-tap OFDM channel estimation setting and enable closed-form MSE expressions while preserving the physical interpretation of MMSE as a Wiener-filtered least-squares estimator.

#### 3.2.1. Zero-Forcing Estimation at Pilot Positions

After Doppler compensation, the received signal at a single pilot position is modeled as

(33) $$y_{p}=H_{p}\cdot P+N_{p}.$$

Using the pilot orthogonality $PP^{H}=E_{p}I$ stated above, the zero-forcing (least-squares) estimator is

(34) $${\hat{H}}_{\mathrm{ZF}}=y_{p}P^{H}/E_{p}=H_{p}+N_{p}P^{H}/E_{p}.$$

The ZF estimate is unbiased ($E\left[{\hat{H}}_{\mathrm{ZF}}\right]=H_{p}$), but it inherits the full noise power of the pilot sample. Under the above pilot-orthogonality assumption, the per-element MSE evaluates to

(35) $${\mathrm{MSE}}_{\mathrm{ZF}}=E\;\left[{\left|{\hat{h}}_{\mathrm{ZF}}-h\right|}^{2}\right]=\frac{\sigma_{n}^{2}}{E_{p}}\underset{E_{p}=1}{\to }\;\frac{\sigma_{n}^{2}}{1}=\frac{1}{\mathrm{SNR}}\sigma_{h}^{2},$$

which decreases only linearly with SNR. In the deep-fade regime (|h|→0 in a given realization), the subsequent ZF equalization step $\hat{x}={\hat{h}}_{\mathrm{ZF}}^{-1}\;y_{d}$ amplifies any noise embedded in ${\hat{h}}_{\mathrm{ZF}}$, motivating the MMSE refinement in Section 3.2.2.

#### 3.2.2. MMSE Filter Optimization

To suppress the noise amplification of the ZF estimate, we apply the Wiener (linear MMSE) filter that minimizes

(36) $$\mathrm{MSE}=E\;\left[\;{\left|h-{\hat{h}}_{\mathrm{MMSE}}\right|}^{2}\;\right].$$

Under the modeling assumptions stated above, the Wiener filter operating on the ZF output ${\hat{h}}_{\mathrm{ZF}}$ is the scalar shrinkage

(37) $${\hat{h}}_{\mathrm{MMSE}}=\frac{\mathrm{Cov}(h,{\hat{h}}_{\mathrm{ZF}})}{\mathrm{Var}({\hat{h}}_{\mathrm{ZF}})}\;{\hat{h}}_{\mathrm{ZF}}=\frac{\sigma_{h}^{2}}{\sigma_{h}^{2}+\sigma_{n}^{2}}\;{\hat{h}}_{\mathrm{ZF}}=\frac{\mathrm{SNR}}{\mathrm{SNR}+1}\;{\hat{h}}_{\mathrm{ZF}},$$

which is the scalar specialization of the general Wiener formulation

(38) $${\hat{H}}_{\mathrm{MMSE}}=R_{H\hat{H}}\;{\left(R_{\hat{H}\hat{H}}\right)}^{-1}\;{\hat{H}}_{\mathrm{ZF}}$$

used in MIMO settings. The corresponding closed-form MSE is

(39) $${\mathrm{MSE}}_{\mathrm{MMSE}}=\frac{\sigma_{h}^{2}\sigma_{n}^{2}}{\sigma_{h}^{2}+\sigma_{n}^{2}}=\frac{\sigma_{h}^{2}}{1+\mathrm{SNR}}.$$

Comparing Equations (35) and (39), the relative MSE improvement of MMSE over ZF is

(40) $$\frac{{\mathrm{MSE}}_{\mathrm{ZF}}}{{\mathrm{MSE}}_{\mathrm{MMSE}}}=\frac{\sigma_{h}^{2}/\mathrm{SNR}}{\sigma_{h}^{2}/\left(1+\mathrm{SNR}\right)}=\frac{1+\mathrm{SNR}}{\mathrm{SNR}}.$$

At low SNR (SNR≪1), the ratio in Equation (40) grows as 1/SNR, i.e., the regularization term $\sigma_{n}^{2}I$ in the Wiener filter dominates and provides large noise suppression. At high SNR (SNR≫1), the ratio approaches 1 and the two estimators converge. In addition, two practical observations follow from Equations (37) and (39). First, because the scalar MMSE estimate differs from the ZF estimate only by a positive real factor, in a one-tap OFDM detector that uses pure phase decision (e.g., QPSK with arg(·) slicer), the resulting symbol decisions are identical and the per-subcarrier BER is the same. The MMSE gain at the BER layer therefore materializes only when coherence-bandwidth pilot averaging is employed to lower $\sigma_{n}^{2}$ at the estimator input, and an MMSE/MRC equalizer of the form

(41) $$\hat{x}=\frac{{\hat{h}}_{\mathrm{MMSE}}^{*}\;y_{d}}{|{\hat{h}}_{\mathrm{MMSE}}{|}^{2}+\sigma_{n}^{2}/N_{\mathrm{corr}}}$$

is used in place of the instantaneous division $y_{d}/\hat{h}$. Here $N_{\mathrm{corr}}$ denotes the number of data subcarriers within one coherence-bandwidth block that share the same MMSE channel estimate ${\hat{h}}_{\mathrm{MMSE}}$. Specifically, in the present configuration with pilot spacing $\Delta f_{p}=16$ subcarriers, the data subcarriers between two adjacent pilots are equalized using the same per-block estimate, giving $N_{\mathrm{corr}}=\Delta f_{p}-1=15$ in the simulations. The role of the term $\sigma_{n}^{2}/N_{\mathrm{corr}}$ is to scale the regularization strength so that, after MRC combining of the $N_{\mathrm{corr}}$ correlated observations, the effective noise floor at the equalizer output is $\sigma_{n}^{2}/N_{\mathrm{corr}}$ rather than $\sigma_{n}^{2}$. Both mechanisms are exploited in the BER experiments of Section 4.2. Second, the absolute MSE floor of MMSE at high SNR is $\sigma_{h}^{2}/\mathrm{SNR}$, which matches the ZF curve in the right tail of Figure 5a within the Monte-Carlo estimation error.

It is therefore useful to attribute the empirical MSE/BER gain over a single-pilot ZF baseline to its three distinct sources, since they enter the receiver chain at different stages. (a) The MMSE estimator itself, viewed as a per-pilot Wiener filter on the ZF output, contributes the shrinkage factor SNR/(SNR+1) in Equation (37). The resulting MSE ratio in Equation (40) gives a low-SNR gain of 1/SNR but only about 3 dB at SNR=0 dB and vanishingly little at SNR≫1. (b) Coherence-bandwidth pilot averaging across $N_{\mathrm{pilot}}$ pilots within one coherence block reduces the effective noise variance by a factor of $N_{\mathrm{pilot}}$, lowering the empirical MSE by $10\log_{10}\left(N_{\mathrm{pilot}}\right)$. For $N_{\mathrm{pilot}}=32$ used in this work, this amounts to about 15 dB and is the dominant contributor to the empirical MSE offset observed in Figure 5a. (c) The MMSE/MRC equalizer in Equation (41) replaces the instantaneous division $y_{d}/\hat{h}$ by a soft inversion, suppressing the noise enhancement in deeply faded subcarriers and converting the residual estimator MSE into a BER gain of 3 to 5 dB at the operating SNR. In other words, the ≈15 dB empirical MSE gap reported in Section 4.2 is not attributable to MMSE versus ZF estimation alone. Rather, it is dominated by source (b) and is realized in BER only through source (c).

## 4. Simulation Parameters and Result Analysis

### 4.1. Simulation Parameter Settings

To evaluate the system model and algorithms presented in Section 2 and Section 3, this section constructs a complete UAV CO-ZP-OFDM simulation platform in MATLAB 2024b. The core simulation parameters are summarized in Table 1. The atmospheric turbulence parameters are derived from the refractive index structure constant $C_{n}^{2}$ through the Rytov variance formula

(42) $$\sigma_{R}^{2}=1.23\;C_{n}^{2}\;k^{7/6}\;L^{11/6},$$

where k=2π/λ is the optical wavenumber, and *L* is the transmission distance. Under typical parameters $C_{n}^{2}=1\times 10^{-14}$ $m^{-2/3}$, L=1 km, λ=1550 nm, we obtain $\sigma_{R}^{2}\approx 0.2255$, and the GG distribution parameters are then α≈11.607, β≈10.094, with scintillation index SI≈0.192. In the pointing-error simulation, the equivalent beam waist radius at the receiver is approximately $w_{\mathrm{eq}}=a\times 1.1547\approx 0.1732$ m (a=0.15 m).

### 4.2. Simulation Results and Analysis

**Figure 3:**
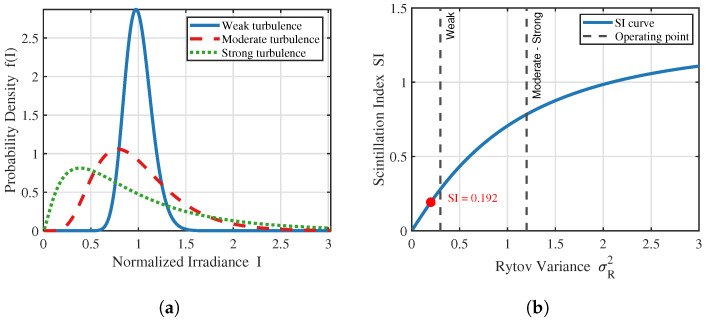
Statistical analysis of the atmospheric turbulence channel. (**a**) GG distribution probability density function. (**b**) Scintillation index vs. Rytov variance.

Figure 3a shows the GG-distribution probability density functions (PDFs) corresponding to three refractive index structure constants ($C_{n}^{2}$). Under weak turbulence ($C_{n}^{2}=10^{-15}$$m^{-2/3}$), the PDF is concentrated near the normalized intensity with a sharp peak and small intensity fluctuation. Under moderate turbulence ($C_{n}^{2}=10^{-14}$$m^{-2/3}$), the PDF peak shifts toward the low-intensity side to about I≈0.85, the overall distribution broadens, and the intensity fluctuation range significantly increases. Under strong turbulence ($C_{n}^{2}=5\times 10^{-14}$$m^{-2/3}$), the PDF peak drops to about I≈0.6, and the tail in the low-intensity region is significant, indicating that the probability of deep fading events increases substantially under strong turbulence.

Figure 3b shows the variation of the scintillation index SI with the Rytov variance $\sigma_{R}^{2}$. The SI increases monotonically. When $\sigma_{R}^{2}<1$ (weak turbulence regime), SI approaches 0. When $\sigma_{R}^{2}>1$ (strong turbulence regime), SI grows slowly and approaches saturation (about 1.2). The red dot marks the operating point of this system ($\sigma_{R}^{2}\approx 0.2255$, SI≈0.192), located at the weak-turbulence boundary, indicating that the intensity fluctuation is about 19.2% of the mean value, which provides reasonable a priori operating conditions for the subsequent MMSE channel estimation.

**Figure 4:**
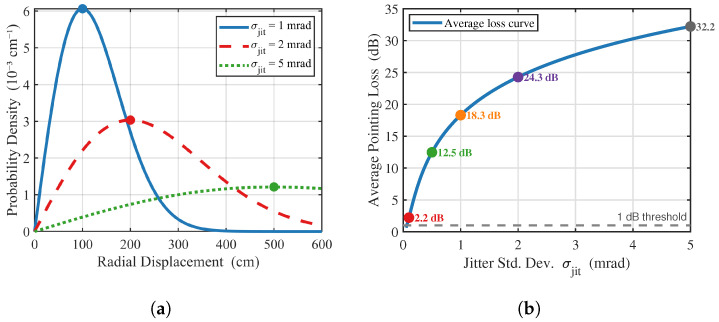
Statistical analysis of pointing-error loss characteristics. (**a**) Radial-offset probability density. (**b**) Average pointing loss vs. jitter std. dev.

Figure 4 presents a statistical analysis of pointing-error loss, quantitatively evaluating the system power cost under different jitter intensities. Figure 4a shows the Rayleigh-distribution PDFs of the radial offset *r* corresponding to three angular jitter standard deviations ($\sigma_{\mathrm{jit}}=1,\;2,\;5$ mrad), where the linear-displacement standard deviation at the receiving plane is $\sigma_{r}=\sigma_{\mathrm{jit}}\times L$. As the jitter increases, the peak of the Rayleigh distribution shifts to the right and decreases in height, indicating a more diffuse offset.

Figure 4b shows the average pointing loss as a function of angular jitter standard deviation, using the Gaussian beam truncation model with $w_{0}=2$ cm and $w_{\mathrm{eq}}\approx 0.1732$ m (consistent with Table 1). The average pointing loss (in dB) is $-10\log_{10}\langle \eta_{p}\rangle$, and the precise values are: $\sigma_{\mathrm{jit}}=0.1$ mrad: loss ≈2.2 dB, $\sigma_{\mathrm{jit}}=0.5$ mrad: loss ≈12.5 dB, $\sigma_{\mathrm{jit}}=1$ mrad: loss ≈18.3 dB, $\sigma_{\mathrm{jit}}=2$ mrad: loss ≈24.3 dB, and $\sigma_{\mathrm{jit}}=5$ mrad: loss ≈32.2 dB. It can be seen that the average pointing loss grows rapidly with $\sigma_{\mathrm{jit}}$. Even a 1 mrad jitter, which is widely regarded as a moderate UAV attitude stability, already incurs more than 18 dB of average power loss at L=1 km. To keep the average pointing loss below 1 dB, the angular jitter must be controlled within $\sigma_{\mathrm{jit}}≲0.062$ mrad (i.e., sub-mrad pointing stability). This implies that pointing errors, together with atmospheric turbulence, dominate the link power budget in UAV A2G FSO scenarios, and high-precision beam-steering or tracking mechanisms are required in practical deployments.

**Figure 5:**
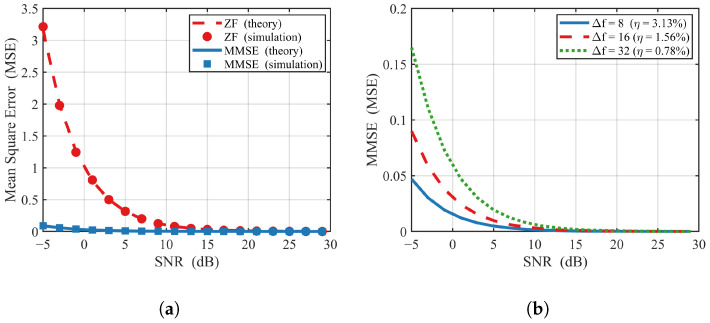
Performance comparison between MMSE and ZF channel estimation. (**a**) Channel estimation MSE: MMSE vs. ZF. (**b**) MMSE MSE under different pilot densities.

Figure 5a compares the theoretical MSE curves of the ZF and MMSE estimators with Monte Carlo simulation points ($N_{\mathrm{MC}}=5000$). The theoretical reference curves are plotted from Equations (35) and (39), which correspond to the single-pilot per-subcarrier estimator. The simulation points, on the other hand, are computed by averaging the squared estimation error across $N_{\mathrm{pilot}}=32$ pilot subcarriers within one coherence block, which is consistent with the PTF pilot structure of Section 2.1. Two observations can be made. First, the simulated ZF MSE curve follows Equation (35) closely. At SNR=0 dB the simulated MSE is about 0 dB (i.e., 1.0), consistent with ${\mathrm{MSE}}_{\mathrm{ZF}}=1/\mathrm{SNR}=1$ predicted by Equation (35). The discrepancy between the theoretical curve and the simulation points stays within ≲0.5 dB across the entire SNR range, consistent with the finite-sample Monte-Carlo variance for $N_{\mathrm{MC}}=5000$. Second, the simulated MMSE MSE is lower than the theoretical single-pilot curve of Equation (39) by an approximately constant offset of $10\log_{10}\left(N_{\mathrm{pilot}}\right)\approx 15$ dB. This offset is not a mismatch but rather an expected averaging gain: when the same Wiener filter is applied across $N_{\mathrm{pilot}}$ pilots within one coherence block, the effective noise variance is reduced by a factor of $N_{\mathrm{pilot}}$, and the empirical MSE decreases accordingly. At SNR=0 dB, the simulated MMSE MSE is about −15 dB (about 0.030), and at SNR=20 dB about −35 dB. This ≈15 dB averaging gain over the single-pilot ZF baseline reflects the noise-averaging mechanism that the PTF pilot grid enables across adjacent pilot subcarriers, which is not available under per-subcarrier ZF. In line with the breakdown given at the end of Section 3.2.2, this ≈15 dB offset should be interpreted primarily as the pilot-averaging factor $10\log_{10}\left(N_{\mathrm{pilot}}\right)$ rather than as a property of the MMSE estimator itself, which contributes only the residual shrinkage term SNR/(SNR+1) shown in the right tail of the figure.

Figure 5b shows the MSE performance curves of the MMSE estimator under three pilot frequency-domain spacings ($\Delta f_{p}=8,\;16,\;32$, corresponding to pilot overheads η=3.13%,1.56%,0.78%). The results show that the higher the pilot density (i.e., the smaller $\Delta f_{p}$), the lower the MSE of MMSE. The MSE gap between adjacent levels is about 3 dB, corresponding to the gain of doubling the number of pilots ($10\log_{10}\left(2\right)\approx 3$ dB). As the SNR increases, the three curves all decrease monotonically and become parallel in the high-SNR region, with the relative gap remaining stable, indicating that pilot density affects the absolute estimation accuracy of MMSE but does not change its rate of decrease with SNR.

**Figure 6:**
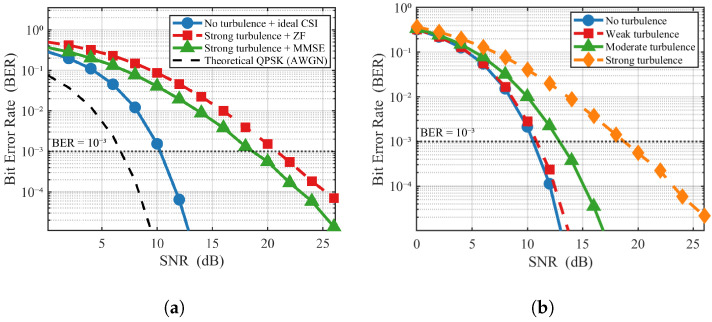
BER performance simulation results of the UAV CO-ZP-OFDM system. (**a**) BER performance vs. SNR. (**b**) BER under different turbulence intensities.

Figure 6a compares the BER curves of the QPSK-modulated system under three channel scenarios versus SNR, with the theoretical QPSK (AWGN) curve as a reference. Three observations can be made. (1) The no-turbulence + ideal-estimation curve closely matches the theoretical QPSK reference, with the BER reaching the $10^{-3}$ level at about 10 dB SNR and falling below $10^{-4}$ at about 12 dB. (2) Under strong turbulence ($C_{n}^{2}=5\times 10^{-14}$ $m^{-2/3}$, SI≈0.70) with ZF equalization (single-pilot LS estimation followed by instantaneous division), the noise amplification of the ZF equalizer at low SNR significantly increases the equivalent error in deeply faded subcarriers, and the BER reaches $10^{-3}$ only at about 22 dB. (3) Under strong turbulence with the proposed MMSE scheme, which combines coherence-bandwidth pilot averaging with the MMSE/MRC equalizer of Equation (41), the MMSE curve reaches $10^{-3}$ at about 18 dB. Compared with the ZF baseline of (2), this corresponds to a 3 to 5 dB shift in the SNR required to attain BER $=10^{-3}$ across the SNR range of practical interest, with the largest gap observed in the mid-SNR region (10 to 22 dB) where deep-fading events dominate the error performance.

Figure 6b, under the MMSE estimation framework, compares the BER performance under four scenarios: no turbulence, weak turbulence ($C_{n}^{2}=10^{-15}$$m^{-2/3}$, SI≈0.02), moderate turbulence ($C_{n}^{2}=10^{-14}$$m^{-2/3}$, SI≈0.19), and strong turbulence ($C_{n}^{2}=5\times 10^{-14}$$m^{-2/3}$, SI≈0.70). Three observations can be made. (1) The no-turbulence and weak-turbulence curves essentially overlap, indicating that weak turbulence has limited impact on the system performance after MMSE equalization. (2) The moderate-turbulence scenario exhibits an SNR degradation of about 2 dB (using $\mathrm{BER}=10^{-3}$ as the reference relative to the no-turbulence baseline). The typical operating point of this system ($C_{n}^{2}=10^{-14}$ $m^{-2/3}$, SI≈0.19) lies in this turbulence range, and the BER reaches the $10^{-3}$ level at about 12 dB SNR. (3) Under strong turbulence, the BER reaches the $10^{-3}$ level at about 18 dB. The deep-fading events of the GG distribution remain the dominant performance bottleneck, but the MMSE estimator with coherence-bandwidth pilot averaging avoids a hard error floor and continues to improve with increasing SNR.

**Figure 7:**
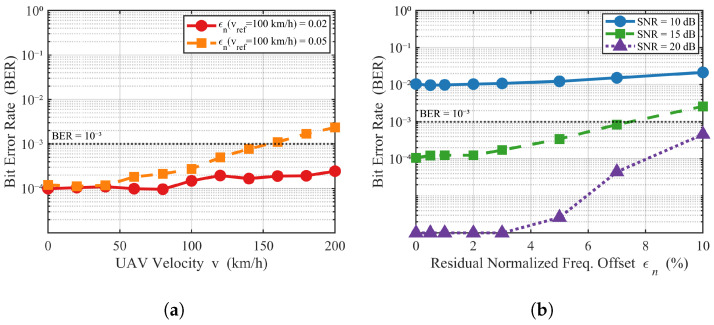
Doppler sensitivity analysis of the proposed CO-ZP-OFDM system under moderate turbulence ($C_{n}^{2}=10^{-14}$ $m^{-2/3}$, SI≈0.19) with MMSE channel estimation and MRC equalization. (**a**) BER vs. UAV velocity. (**b**) BER vs. residual normalized frequency offset.

To address the Reviewer’s concern that the motivation of this paper emphasizes the UAV mobility and high Doppler shift but the previous figures do not quantify their impact, a dedicated Doppler sensitivity study is provided in Figure 7. The analysis is conducted on top of the proposed MMSE channel estimator with coherence-bandwidth pilot averaging. The residual Doppler effect is injected through the well-known inter-carrier-interference (ICI) model of Pollet et al.: a residual normalized frequency offset $\epsilon_{n}=\epsilon_{f}/\Delta f$ raises the effective noise variance by a factor of $1+\left(\pi^{2}/3\right)\epsilon_{n}^{2}{\mathrm{SNR}}_{\mathrm{lin}}$, where $\epsilon_{f}$ is the residual Doppler frequency in Hz and Δf is the OFDM subcarrier spacing.

Figure 7a shows the BER as a function of UAV velocity for two estimator accuracy classes, characterized by the residual normalized frequency offset at a reference velocity of 100 km/h, namely $\epsilon_{n}\left(v_{\mathrm{ref}}\right)=0.02$ and 0.05. The residual NFO is scaled linearly with velocity to reflect the upper bound of the Doppler estimation accuracy in practical implementations. At the working SNR of 15 dB, with the more accurate algorithm ($\epsilon_{n}\left(v_{\mathrm{ref}}\right)=0.02$), the BER grows mildly with velocity, from about $7\times 10^{-5}$ at rest to about $2\times 10^{-4}$ at 200 km/h, well below the $10^{-3}$ target. With the less accurate algorithm ($\epsilon_{n}\left(v_{\mathrm{ref}}\right)=0.05$), the BER deteriorates by an order of magnitude over the same velocity range, reaching approximately $2.5\times 10^{-3}$ at 200 km/h, which exceeds the $10^{-3}$ threshold. The two curves bracket the BER range corresponding to typical Doppler-tracking accuracies in practical implementations.

Figure 7b shows the BER as a function of the residual NFO $\epsilon_{n}$ at three working SNR points (10, 15, and 20 dB) with the velocity fixed at 100 km/h. The figure reveals a clear SNR-dependent sensitivity. At low SNR (10 dB), the BER floor is dominated by the AWGN and turbulence and the Doppler residual has limited additional impact. At moderate SNR (15 dB), the BER rises noticeably from $9\times 10^{-5}$ at $\epsilon_{n}=0$ to about $2\times 10^{-3}$ at $\epsilon_{n}=0.10$, a 22-fold increase. At high SNR (20 dB), the system becomes most sensitive, with an effective error floor appearing at $\epsilon_{n}\approx 0.10$. The implication for system design is that, to keep the BER below $10^{-3}$ at the proposed operating point of 15 dB SNR, the residual normalized frequency offset must be kept below approximately 5%, which corresponds to an absolute Doppler-tracking accuracy of about 5 kHz for the 100 kHz subcarrier spacing used in this work.

## 5. Conclusions

This paper develops a CO-ZP-OFDM transmission framework tailored to UAV air-to-ground optical backhaul, which jointly models atmospheric turbulence scintillation, pointing-error loss, and Doppler frequency shift on a polarization-time-frequency sparse pilot grid. A closed-form theoretical comparison between ZF and MMSE estimators is established under the stated assumptions, with the MSE of ZF decreasing inversely with SNR while MMSE introduces an additional regularization term. The MMSE estimator itself contributes only the shrinkage factor SNR/(SNR+1) and yields a per-pilot MSE gain that is significant only at low SNR. However, the empirical ≈15 dB reduction in MSE observed in Section 4.2 is dominated by the pilot-averaging factor $10\log_{10}\left(N_{\mathrm{pilot}}\right)\approx 15$ dB across $N_{\mathrm{pilot}}=32$ pilots within one coherence block, while the subsequent 3 to 5 dB shift in the BER curve relative to the ZF baseline under strong turbulence is realized by the MMSE/MRC equalizer of Equation (41). At the typical operating point corresponding to moderate turbulence with scintillation index around 0.19, the system reaches a BER of $10^{-3}$ at about 12 dB SNR, while the no-turbulence and weak-turbulence cases give nearly identical curves. The Doppler sensitivity analysis indicates that, for a subcarrier spacing of 100 kHz, the residual normalized frequency offset must be kept below approximately 5% to maintain a BER below $10^{-3}$ at 15 dB SNR, which corresponds to an absolute Doppler tracking accuracy of about 5 kHz. The updated pointing-error analysis shows that sub-mrad pointing stability with angular jitter below 0.062 mrad is required to keep the average power loss within 1 dB at a 1 km link, indicating that pointing errors are a non-negligible loss factor alongside atmospheric turbulence in UAV A2G optical links. In addition, the present analysis is restricted to single-link quasi-static channels with one-tap scalar estimation. Future work will extend the framework to multi-UAV cooperative links with joint Doppler and pointing tracking, adaptive modulation and coding under time-varying turbulence, and deep-learning-aided non-linear MMSE estimators for the full 2×2 Jones-matrix channel.

## Figures and Tables

**Table 1:** Summary of system simulation parameters.

Parameter	Symbol	Value	Unit
Carrier wavelength	λ	1550	nm
Optical wavenumber	k=2π/λ	$4.05\times 10^{6}$	rad/m
Transmission distance	*L*	1	km
Refractive index structure constant	$C_{n}^{2}$	$1\times 10^{-14}$	$m^{-2/3}$
Rytov variance	$\sigma_{R}^{2}$	0.2255	N/A
GG turbulence parameters	α,β	11.607, 10.094	N/A
Scintillation index	SI	0.192	N/A
Transmit beam waist radius	$w_{0}$	0.02	m
Receive aperture radius	*a*	0.15	m
Equivalent beam waist radius	$w_{\mathrm{eq}}$	0.1732	m
Angular jitter std. deviation	$\sigma_{\mathrm{jit}}$	1/2/5	mrad
FFT/IFFT size	$N_{\mathrm{FFT}}$	512	N/A
Number of active subcarriers	$N_{\mathrm{sub}}$	128	(centred in FFT)
Zero-padding length	*M*	64	samples
Subcarrier spacing	Δf	100	kHz
OFDM symbol duration	$T_{\mathrm{sym}}$	11.25	μs
Modulation scheme	N/A	QPSK	N/A
Number of simulated symbols	$N_{\mathrm{sym}}$	4000	N/A
SNR range	SNR	0∼26	dB
Pilot spacing	$\Delta f_{p}$	8/16/32	subcarriers
Channel gain variance	$\sigma_{h}^{2}$	1.0	N/A
Number of MC simulations	$N_{\mathrm{MC}}$	5000	N/A

## Data Availability

Data are contained within the article.

## References

[1] An N., Yang F., Cheng L., Song J., Han Z. (2023). Free space optical communications for intelligent transportation systems: Potentials and challenges. IEEE Veh. Technol. Mag..

[2] Zaid A.A., Belmekki B.E.Y., Alouini M.-S. (2023). eVTOL communications and networking in urban air mobility: Requirements, key enablers, and challenges. IEEE Commun. Mag..

[3] Zheng Y., Li L., Lin W., Liang W., Du Q., Han Z. (2026). Optimal Transport Framework for ISAC in Low-Altitude Networks: Joint Resource Allocation for Cooperative Communication and Non-Cooperative Localization. IEEE Trans. Commun..

[4] Wang M., Wang D., Zhao H., He Y., Tang X., Zhou F., Wei Z., Li L. (2026). Cognitive Spectrum Sharing in Space-Air-Ground Integrated Networks: Optimizing Beamforming and AAV Trajectories with Two-Layer RSMA. IEEE Trans. Cogn. Commun. Netw..

[5] Hu B., Zhang W., Yin Y., Du J., Yu F.R., Chu X. (2026). Soft Actor-Critic-Based Offloading and Resource Allocation Optimisation for Digital Twin-Aided Vehicular Networks. IEEE Trans. Veh. Technol..

[6] Zhao H., Wang Z., Zhuang C., Zhao Q. (2026). Outlier mitigation for cooperative positioning using graduated nonconvexity truncated least-squares cost. IEEE Trans. Instrum. Meas..

[7] He Y., Huang F., Wang D. (2026). PP-RCR: A position-probability-resource cooperative routing algorithm for air-to-ground integrated mobile ad hoc networks in emergency rescue scenarios. IEEE Wirel. Commun. Lett..

[8] Liu X., Gao Z., Wan Z., Wu Z., Li T., Mao T., Liang X., Zheng D., Zhang J. (2025). Toward near-space communication network in the 6G and beyond era. Space Sci. Technol..

[9] Donipati M., Jaiswal A., Hazra A., Mazumdar N., Singh J. (2025). Optimizing UAV-Based Data Collection in IoT Networks with Dynamic Service Time and Buffer-Aware Trajectory Planning. IEEE Trans. Netw. Serv. Manag..

[10] Hu B., Du J., Zhang J., Chu X. (2025). Computation Offloading and Resource Allocation in Mixed Cloud/Vehicular-Fog Computing Systems. IEEE Trans. Mob. Comput..

[11] He Y., Huang F., Liang Y., Wang D., Zhao H., Lou J., Zhang R. (2026). Sum Secrecy Rate Enhancement in Low-Altitude Intelligent Networks with Mixed Obstacles. IEEE Internet Things J..

[12] Li J., Wang D., Zhang H., Jin Y., He Y., Zhou F., Wei Z., Leung V.C.M. (2026). Enhancing Secrecy Energy Efficiency in UAV-RIS Assisted Mobile IoV Networks Through DRL. IEEE Trans. Wirel. Commun..

[13] Saif M., Valaee S. (2025). RIS Alignment via Virtual Partitioning for Resilient Uplink Multi-RIS-Assisted UAV Communications. IEEE Trans. Commun..

[14] He S., Wang J., Liang Y.-C., Sun G., Niyato D. (2025). Satellite-assisted low-altitude economy networking: Concepts, applications, and opportunities. IEEE Commun. Surv. Tutor..

[15] Ning Z., Li T., Wu Y., Wang X., Wu Q., Yu F.R., Guo S. (2025). 6G communication new paradigm: The integration of unmanned aerial vehicles and intelligent reflecting surfaces. IEEE Commun. Surv. Tutor..

[16] Zhao H., Wu M., Liu T., Zhang D., Wang D., Liu R. (2025). Federated learning-based ISAC network in cohesive clustered satellite: Resource optimization in heterogeneous datasets and systems. Sci. China Inf. Sci..

[17] Hu B., Liu H., Cao H., Yuan S., Chu X., Xu S. (2026). Digital Twin-Assisted Large AI Task-Aware Edge Offloading and Resource Allocation for Low-Altitude Wireless Sensor Networks. IEEE J. Sel. Areas Sens..

[18] Wu M., Lai S., Lu W., Zhao B., Guo L., Nallanathan A. (2026). Rethinking Fluid Antennas for ISAC: A Systematic Review and a Novel Air–Ground Collaborative Framework. IEEE Trans. Netw. Sci. Eng..

[19] Huang J., Wu B., Duan Q., Dong L., Yu S. (2025). A Fast UAV Trajectory Planning Framework in RIS-Assisted Communication Systems With Accelerated Learning via Multithreading and Federating. IEEE Trans. Mob. Comput..

[20] Wu M., Wu H., Lu W., Guo L., Lee I., Jamalipour A. (2025). Security-Aware Designs of Multi-UAV Deployment, Task Offloading and Service Placement in Edge Computing Networks. IEEE Trans. Mob. Comput..

[21] Wu M., Gao Y., Song Q., Li K., Lu W., Guo L., Jamalipour A. (2025). Integrated Resource Collaboration for RIS-Assisted Digital-Twin-Empowered Internet of Everything. IEEE Internet Things J..

[22] Zhao H., Wu M., Wang D., Guizani M., Leung V.C.M. (2026). Sensing-assisted secure beamforming for RIS-enabled ISAC with leakage suppression. IEEE Trans. Wirel. Commun..

[23] Wang D., Li J., Lv Q., He Y., Li L., Hua Q., Alfarraj O., Zhang J. (2025). Integrating reconfigurable intelligent surface and AAV for enhanced secure transmissions in IoT-enabled RSMA networks. IEEE Internet Things J..

[24] Gupta A., Dhawan D., Gupta N. (2024). Review on UAV-based FSO links: Recent advances, challenges, and performance metrics. Opt. Eng..

[25] Khalighi M.A., Uysal M. (2014). Survey on free space optical communication: A communication theory perspective. IEEE Commun. Surv. Tutor..

[26] Kaushal H., Kaddoum G. (2017). Optical communication in space: Challenges and mitigation techniques. IEEE Commun. Surv. Tutor..

[27] Andrews L.C., Phillips R.L. (2005). Laser Beam Propagation Through Random Media.

[28] Sharma D., Tripathi A., Kumari M. (2024). FSO systems for next generation networks: A review, techniques and challenges. J. Opt. Commun..

[29] Alimi I.A., Monteiro P.P. (2024). Revolutionizing free-space optics: A survey of enabling technologies, challenges, trends, and prospects of beyond 5G free-space optical communication systems. Sensors.

[30] Hassan H., Althunibat S., Al-Mbaideen A., Hasna M., Qaraqe K.A. (2025). A survey on hybrid free space optical and radio frequency systems: Classification, progress, observations, and challenges. IEEE Access.

[31] Al-Habash M.A., Andrews L.C., Phillips R.L. (2001). Mathematical model for the irradiance probability density function of a laser beam propagating through turbulent media. Opt. Eng..

[32] Tarimo C., Kulkarni M. (2023). Performance analysis of relay-assisted FSO communication over Gamma-Gamma atmospheric turbulence channels with pointing errors. Proceedings of the IIWCS.

[33] Farid A.A., Hranilovic S. (2007). Outage capacity optimization for free-space optical links with pointing errors. J. Light. Technol..

[34] Badarneh O.S., Bouanani F.E., Almehmadi F.S., Silva H.S. (2023). FSO communications over doubly inverted gamma-gamma turbulence channels with nonzero-boresight pointing errors. IEEE Wirel. Commun. Lett..

[35] Zhang S., Tan L., Ma J. (2023). Flexible phase synchronization for wireless optical coherent communication system with adaptive fractionally-spaced blind equalization combined with adaptive Kalman filter. IEEE Photon. J..

[36] Zhang Q., Liu B., Chen G., Zhan S., Li Z., Zhang J., Jiang N., Cao B., Li Z. (2024). An improved adaptive coding and modulation scheme with hybrid switching standard for UAV-to-ground free space optical communication. IEEE Photonics J..

[37] Saeed M., Eldemerdash Y.A., El-Malek A.H.A., Zummo S.A. (2024). A comprehensive review of UAV-assisted FSO relay systems. Photonics.

[38] Shieh W., Athaudage C. (2006). Coherent optical orthogonal frequency division multiplexing. Electron. Lett..

[39] Shieh W., Bao H., Tang Y. (2008). Coherent optical OFDM: Theory and design. Opt. Express.

[40] Shao Y., Yu N., Wang A., Tian Q., Yi L., Yang Q., Li Y., Liu S., Yuan J., Zuo R. (2024). Research on full duplex FSO access system with hybrid 16PSK/256QAM-OFDM downlink and duobinary uplink signals. Opt. Commun..

[41] Belgaonkar V.V., Sundaraguru R., Poongothai C. (2024). Free space optical communication using OQPSK in the presence of strong atmospheric turbulence and losses. Opt. Quantum Electron..

[42] Muquet B., Wang Z., Giannakis G.B., de Courville M., Duhamel P. (2002). Cyclic prefixing or zero padding for wireless multicarrier transmissions. IEEE Trans. Commun..

[43] Lv P., Hong Y. (2023). Self-pilot tone based adaptive threshold RZ-OOK decision for free-space optical communications. Photonics.

[44] Rani R., Kaur G. (2024). Performance analysis of free space optical system over inverse Gaussian gamma turbulence channel. Trans. Emerg. Telecommun. Technol..

[45] Shakir W.M., Mahdi A.S., Hamdan H., Charafeddine J., Al Satai H., Akrache R., Haddad S., Sayah J. (2024). Novel approximate distribution of the generalized turbulence channels for MIMO FSO communications. IEEE Photonics J..

[46] Sharma P., Swaminathan R., Singh D. (2024). Multi-Hop UAV-Based FSO System Over Doubly Inverted Gamma-Gamma Turbulence Channel. IEEE Commun. Lett..

[47] Gökçe M.C., Ata Y., Baykal Y. (2022). Performance evaluation of aeronautical uplink/downlink free-space optical communication system with adaptive optics over gamma-gamma turbulence channel. J. Opt..

[48] Ishaq I., Jamel T.M. (2026). Improving the performance of Gamma-Gamma FSO links with adaptive optics, dynamic beam steering, and strong modulation formats. J. Opt. Commun..

[49] Liu X., Buchali F. (2008). Intra-symbol frequency-domain averaging based channel estimation for coherent optical OFDM. Opt. Express.

[50] Shahmohammadi M., Sebghati M., Zareian H. (2024). Deep learning-based pilot adaptation and channel estimation in OFDM systems. Wirel. Pers. Commun..

[51] Lapsiwala P.B., Vasava P.B. (2024). Link handling for the atmospheric turbulence using LSTM neural networks in free space optical (FSO) communication. J. Opt. Commun..

[52] Kavitha M., Geetha M., Rajesh T. (2025). Mitigating the impact of lognormal atmospheric turbulence channel estimation on FSO communication systems using advanced deep learning modules. Int. J. Commun. Syst..

[53] Senthilkumar S., Balakrishnan R., Irshad Ahamed M., Senthil Kumar T. (2026). A sparse wavelength aware learning network for robust FSO channel estimation. Sci. Rep..

[54] Prasanna A.D., Das S.K. (2025). Machine learning-based channel estimation for free space optical (FSO) communication. Proceedings of the IIWCS 2024.

[55] Chen D., Wang R., Wang C., Gao Y., Chen H. (2024). Joint estimation model for FSO channel parameters and performance evaluation based on CNNs. Appl. Opt..

[56] Ahmad M., Hayat B., Fang M., Wang C., Xie G., Huang Z. (2026). Robust high-capacity free-space optical communication using OAM-based structured light and intelligent adaptive signal processing. Sci. Rep..

[57] Ishaq I., Jamel T.M. (2026). Sequence-aware OOK detection in turbulent FSO using bidirectional GRU/LSTM. J. Opt. Commun..

